# Navigating the ventricles: Novel insights into the pathogenesis of hydrocephalus

**DOI:** 10.1016/j.ebiom.2022.103931

**Published:** 2022-03-17

**Authors:** Alexa N. Bramall, E.S. Anton, Kristopher T. Kahle, Peter E. Fecci

**Affiliations:** a Department of Neurosurgery, Duke University Hospital, 2301 Erwin Rd., Durham, NC 27710, United States; b UNC Neuroscience Center and the Department of Cell Biology and Physiology, University of North Carolina School of Medicine, Chapel Hill, NC, United States; c Department of Neurosurgery, Massachusetts General Hospital, Boston, MA, United States

**Keywords:** Hydrocephalus, Choroid plexus, Ependymal cells, Glymphatic system

## Abstract

Congenital hydrocephalus occurs in one in 500–1000 babies born in the United States and acquired hydrocephalus may occur as the consequence of stroke, intraventricular and subarachnoid hemorrhage, traumatic brain injuries, brain tumors, craniectomy or may be idiopathic, as in the case of normal pressure hydrocephalus. Irrespective of its prevalence and significant impact on quality of life, neurosurgeons still rely on invasive cerebrospinal fluid shunt systems for the treatment of hydrocephalus that are exceptionally prone to failure and/or infection. Further understanding of this process at a molecular level, therefore, may have profound implications for improving treatment and quality of life for millions of individuals worldwide. The purpose of this article is to review the current research landscape on hydrocephalus with a focus on recent advances in our understanding of cerebrospinal fluid pathways from an evolutionary, genetics and molecular perspective.

## Introduction

Hydrocephalus, or “*hydro”* and “*kephal”* meaning “water on the brain” was noted in skeletal descriptions dating from 2500 BC to 500 AD. Historically, hydrocephalus has been divided into communicating versus non-communicating, and congenital versus acquired. Acquired forms can result from a plethora of environmental insults such as traumatic brain injuries, tumors, cysts, hemorrhagic and ischemic stroke. The term hydrocephalus often implies increased intracranial pressure (ICP) as dictated by the Monroe-Kellie doctrine, but is not necessarily synonymous with elevated ICP, as in conditions like normal pressure hydrocephalus (NPH) and low-pressure hydrocephalus. While the earliest use of the term hydrocephalus has been attributed to Hippocrates (466-377BC),^1^ cerebrospinal fluid (CSF) was first defined by Galen of Pergamon (130-200AD) whom referred to CSF as a “vaporous humor in the ventricles that provided energy to the entire body”, or the *spiritus animalus* in Latin.^1^

### CSF: a developmental and evolutionary perspective

Although there has been much speculation as to why human brains contain CSF reservoirs, the exact purpose is still unclear. The existence of a clear fluid around brain tissue was first scientifically demonstrated by Cotugno in the 18th century and Francois Magendie from 1825–1842, for which the midline foramen in the fourth ventricle was named, also termed the metapore. Luschka then derived the name of the lateral recesses of the fourth ventricle in 1854 and Key and Retzius expounded on the main structures of the pia, arachnoid and dura mater in 1875. Interestingly, although a metapore was noted in the amphibian newt *Diamectylus viridescens* by Gage in 1893, Blake in 1900 reported the presence of the metapore only in humans and three Old World monkey species and absent in all other species he examined.^2^

**Figure 1:**
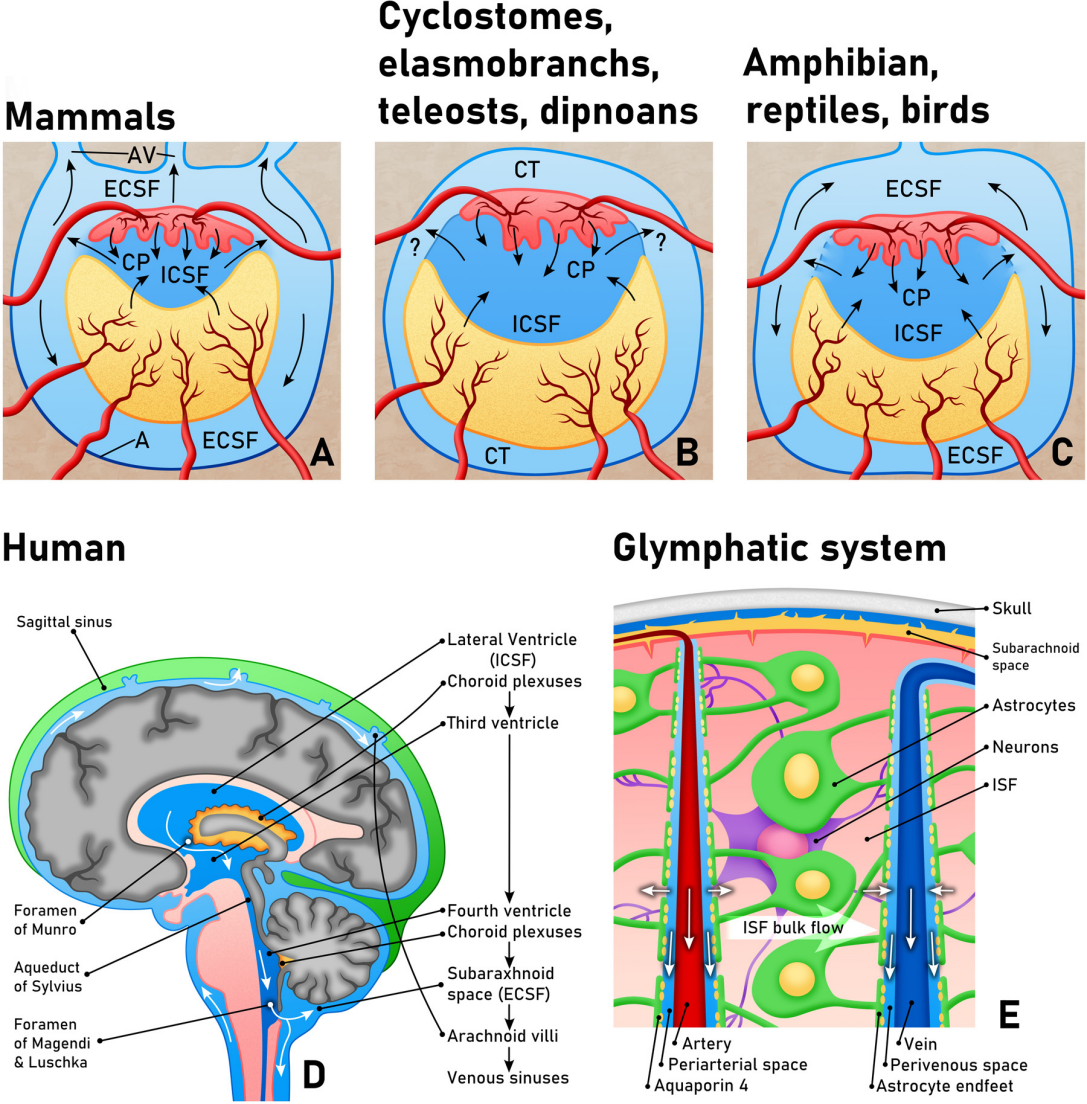
Evolutionary relationships of CSF and the glymphatic system. A. In mammals, the internal CSF (ICSF) is secreted by the choroid plexus (CP) and is continuous with the external CSF (ECSF) or subarachnoid space via the foramina of the fourth ventricle. B. In cyclostomes, elasmobranchs, teleosts and dipnoans, the ICSF is not in communication with an ECSF compartment but may be able to pass into the pericerebral connective tissue (CT). C. In amphibians, reptiles and birds, there is an absence of a metapore or foramen, however, ICSF is thought to pass through a permeable membrane to reach the ECSF compartment. Note that absorption through arachnoid villi (AV) only occurs in certain mammalian species, as shown in A. Modified from.^64^ D. CSF circulation in humans, and a closer image of the glymphatic system (E) showing the flow of fluid within the interstitial compartment from periarterial to perivenous spaces in the brain. Astrocytic endfeet make up a significant part of the blood brain barrier and aquaporin-4 has been shown to regulate extracellular fluid volume.

Cushing later helped to further develop the canonical view of CSF flow from the choroid plexus to the subarachnoid space and venous sinuses; however, this theory is notably inconsistent with other species lacking a metapore. Further examination of CSF morphology across species reveals that a connection between the hindbrain linking the ventricles to an external CSF compartment is not consistent across species; some groups of fish including elasmobranchs, teleosts, cyclostomes and dipnoans do not contain any external CSF compartment (Figure 1). These CSF systems contain cerebral ventricles lined by ependymal cells and choroid plexus and the space immediately surrounding the brain contains loose connective tissue with extradural fluid which does not associate with the intraventricular compartment.^3^ The meninges in these animals are also different, with an absence of an arachnoid membrane. Scientists consequently hypothesized that CSF absorption in these species may occur through the ependymal cells or via contact with the perimeningeal spaces through perivascular spaces in brain parenchyma.

CSF may also play a role in the normal development of the central nervous system (CNS). First, increases in embryonic CSF volume exerts a positive pressure against the neuroepithelial walls during development and generates an expansive force, contributing to brain expansion and neurogenesis.^4^ Interestingly, repeatedly draining CSF from embryonic zebrafish brain ventricles prevents brain enlargement and also decreases the rates of neuroepithelial cell survival.^5^

Embryonic CSF also contains a number of growth factors and cytokines which contribute to the regulation of basic neural progenitor cell functions including survival, proliferation and differentiation.^6^ Moreover, the effect of sampled CSF on neural progenitor cells depends on the developmental stage from which the fluid has been obtained, indicating that CSF may contain developmentally specific regulators. Many of these developmentally important growth factors, such as FGF2, may derive from the embryonic environment prior to closure of the anterior neuropore.^7^ The choroid plexus epithelium also secretes IGF-II, with continued secretion of factors such as EGF, FGF-2 and retinoic acid in adults, which are important for neurogenesis in the subventricular zone.^8^

Aside from the role of CSF in normal brain development, it is also speculated to act as a cushioning system and buoyant force which reduces the overall weight of the brain from 1500 to 25–50 g.^9^ This theory has been used to partially explain the appearance of an external CSF compartment in terrestrial animals as more cushioning of the brain was required in the transition from a liquid to gaseous environment. This increased buoyancy also helps to protect the brain against impact, cushioning the brain and spinal cord from the bony skull and spinal column. Although this theory is enticing, it nonetheless this does not explain the presence of CSF anatomy comparable to humans in marine mammals such as dolphins and whales.

### The biological and chemical composition of CSF

The composition of CSF is similar to blood serum except chloride concentrations are higher and the potassium and calcium concentrations significantly lower,^10^ with little protein. The unique composition of CSF is especially important because perturbations may indicate a pathologic process. For example, glucose concentrations in bacterial meningitis decrease to < 0.4 of serum levels, and using a cutoff value of 0.36, the sensitivity and specificity of the ratio of glucose in spinal fluid to serum is 93%.^11^^,^^12^ Comparatively, the classic clinical triad of fever, neck stiffness and altered mental status has only a 40–50% sensitivity for the diagnosis of bacterial meningitis. Increases in CSF sodium concentration in during migraine, has been documented in multiple studies and may relate to perturbations in brain, blood and CSF barriers.^13^ CSF biomarkers have also been identified for multiple sclerosis, Parkinson's disease and Alzheimers disease (AD).^14^^,^^15^ AD is probably the most extensively studied, and in AD, the ratio of the content of amyloid beta peptide Aβ_42/_ Aβ_40_ has been used to predict cortical amyloid deposition.^16^ Idiopathic normal pressure hydrocephalus (iNPH) may also show changes in Aβ_42_ levels compared with age-matched controls.^17^

### CSF production and absorption

**Figure 2:**
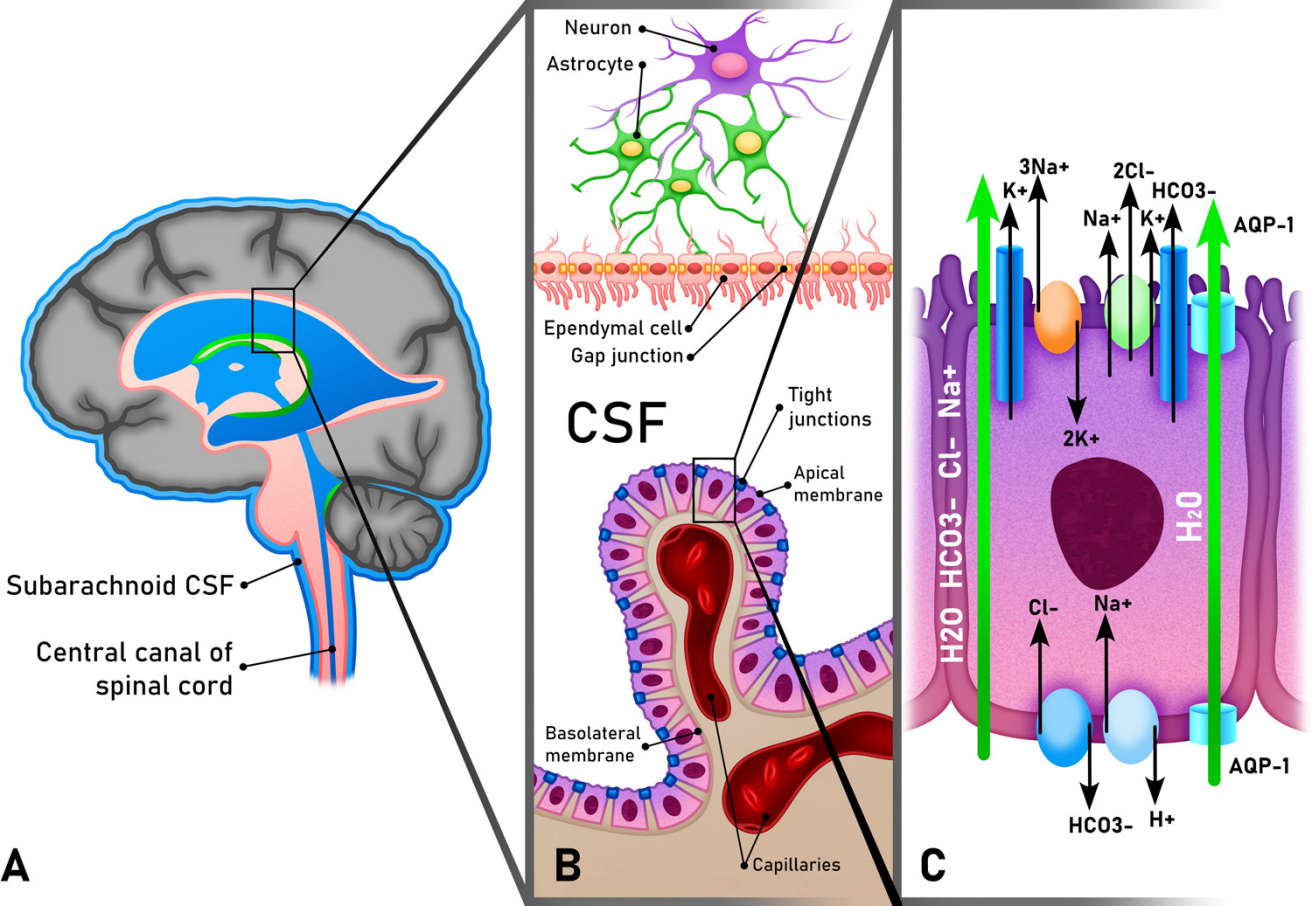
Choroid plexus epithelial cells and ventricular ependymal cells. A. General sketch of CSF flow within the human brain with magnification of the choroid plexus and ventricular ependymal cells in B. The choroid plexus is composed of modified ependymal cells with tight junctions and a stromal layer with fenestrated capillaries. This layer is continuous the ependymal cells lining the ventricle, which are connected via gap junctions. The apical membrane of choroid plexus epithelium faces the internal CSF compartment. C. Important channels in the apical and basolateral membranes of choroid plexus epithelial cells. NKCC1 is predominantly located on the apical membrane, where it plays an important role in the production of CSF through the activation of aquaporin-1 channels, which have been found both on apical and basolateral membranes but predominantly on the apical side of CPECs.

The choroid plexus is thought to be responsible for 70–80% of CSF production. The term originally derived from the Latin for “chorion,” or delicate, with “plexus” based on its vascular architecture. Indeed, all vertebrate species share a choroid plexus, and in human embryos, the choroid plexus accounts for over 60% of the total ventricular surface, signifying an important and evolutionarily conserved function. The choroid plexus receives more blood per gram of tissue than any other part of the brain. It is composed of an interstitial stromal layer, a fenestrated vascular core, and a single layer of polarized cuboidal epithelium or modified ependymal cells joined by tight junctions (Figure 2). These choroid plexus epithelial cells (CPECs) are contiguous with the ependymal cells that line the ventricular cavities and the spinal canal (sometimes termed ependymocytes). Unlike the CPECs, ependymal cells do not have tight junctions but rather communicate via adherens and gap junctions, the latter of which have been implicated in the normal synchronization of ciliary beating and CSF circulation.^18^

Both CPECs and ependymal cells contain cilia, although they differ in structure. CPECs have between one to two dozen primary cilia, whereas ependymal cells have hundreds of motile cilia equipped with axonemes containing a central pair of microtubules in the 9+2 configuration (versus 9+0 for primary cilia). Although primary cilia are typically non-motile, they still contain receptors with the capacity to respond to mechanical and chemical stimuli.^19^ Additionally, there is some evidence that the cilia of CPECs may show transient motility in the neonatal period, although not sufficient to produce directional fluid flow.^20^

The remainder of CSF production is believed to come from ependymal cells lining the ventricles and in the subarachnoid space as well as fluid transport across the blood brain barrier (BBB), formed by the tight junctions of endothelial cells along cerebral capillaries. Fluid that is transported across the BBB becomes interstitial fluid (ISF) which includes water-soluble metabolites making up the extracellular space between neurons and glia in the parenchyma. CSF and ISF then communicate in the pericapillary spaces (Virchow robin spaces) across the pia as well as through the ependymal lining^9^^,^^21^ (Figure 2). The cephalopod brain (belonging to squid, octopus and cuttlefish) is especially instructive as these animals have undergone more than 500 million years of evolution independent from humans. They have nervous systems that are the most complex of all invertebrates and a correspondingly high brain to body ratio.^22^ These animals have an ISF flow rate similar to that of the rat as well as a similar BBB but no choroid plexus, ventricles, or CSF, suggesting that all ISF in these brains must arise from capillary secretion.^23^

As previously mentioned, Cushing helped to develop the canonical view of CSF flow from the choroid plexus to the subarachnoid space through the foramen of Luschka and Magendie, and then to the cerebral venous sinuses, through which it is absorbed via arachnoid granulations. Arachnoid granulations or arachnoid villi (on a microscopic level) are outpouchings of arachnoid mater located along the venous sinuses and intercavernous sinuses in the skull and have classically been considered the cells responsible for the absorption of CSF in a one-way valve-like type flow mechanism. At birth, however, arachnoid granulations are not fully present, and the absorption of CSF is believed to occur through the venous plexus of the inner surface of the dura, which is more robust in infants.^24^ Additionally, it is well known that mouse, rat, rabbit, and cats do not have arachnoid granulations through their entire lifespan,^25^ and many species do not even harbor an external CSF compartment, as previously discussed.

Historically, the brain was believed to be an immune privileged environment due to the lack of a canonical lymphatic system. However, recent evidence has shown that the brain has an alternative to a traditional lymphatic system which involves multiple pathways of CSF circulation and egress for the maintenance of fluid homeostasis as well as immune surveillance. First, CSF may flow along olfactory and spinal nerves to the cervical and spinal lymphatics, and experiments whereby drainage from the cribriform plate was blocked after isolation of cranial from spinal lymphatics, led to increases in resting ICP in a sheep model.^26^ Second, the CSF and ISF compartment within the brain parenchyma may communicate through paravascular networks along penetrating arteries and draining veins, which enables the bidirectional exchange of fluids and solutes. This glymphatic system is controversial but appears to be dependent on the astroglial water channel aquaporin-4 (AQP4),^27^ which is found on the endfeet of astrocytes surrounding cerebral vasculature, an important component of the BBB. Third, there is evidence of a robust meningeal lymphatic system with a network of lymphatic vessels running alongside the dural venous sinuses, draining into the deep cervical lymph nodes.^28^ Although there are lymphatic vessels along the dorsal venous sinuses, the meningeal lymphatic vessels in the basal and lateral parts of the skull have been shown to be predominantly involved in the drainage of tracers from the CSF to the deep and superficial cervical lymph nodes.^29^

## Mechanisms of hydrocephalus

### CSF flow dynamics

Theoretically, hydrocephalus may be caused by any mechanism that disrupts the equilibrium between CSF absorption and production. This could include, for instance, the hypersecretion of CSF at the level of the choroid plexus or the blockage of CSF absorption at the level of the arachnoid villi. The explanation in these situations is direct and mechanical. In fact, the presence of blood within the ventricular system after intraventricular hemorrhage (IVH), one of the leading causes of secondary, acquired hydrocephalus, has been thought of as a direct form of outlet obstruction, causing blood clots to obstruct the drainage of CSF through the arachnoid villi. Additionally, inflammation from blood products in the intraventricular space, meningitis, or any other type of pro-inflammatory signal can cause fibrosis and scarring, serving as another mechanical barrier to normal CSF flow. Although hydrocephalus may form in the acute phase, due to direct mechanical obstruction, a delayed reaction to inflammatory blood breakdown products is also the means by which hydrocephalus may occur in a delayed fashion and become chronic.^30^

This concept of hydrocephalus as a simple disequilibrium in production and absorption is based on a bulk flow model of CSF flow proposed by Harvey Cushing in 1925.^31^ Although the simplistic view of CSF directional flow from the choroid plexus and ventricular system to the subarachnoid space is generally accepted, there are many nuances to CSF flow dynamics that are not well understood and/or appreciated. More recent experiments in mice indicate that the flow of CSF in perivascular spaces is driven by arterial oscillations due to the cardiac cycle, with the respiratory cycle as a secondary driver. Hypertension then significantly slows transport in perivascular spaces due to increased backflow from pulsations, and reduced netflow.^32^ These systolic pressure waves are also transmitted to the subarachnoid space and venous capacitance vessels and interact with intraventricular pulsations transmitted by the choroid plexus. Decreased compliance may thus cause abnormally high pulsation amplitudes resulting in ventricular expansion.^32^ Any mechanism via which compliance is altered, therefore, common after any type of brain injury, may influence normal CSF flow dynamics. Additionally, inflammation within the intracranial compartment and associated scarring can cause a decrease in compliance, again leading to disturbances of CSF flow and ventricular expansion.

### The role of transport proteins

Transport proteins are critical in maintaining homeostasis in the brain through the transport of ions between intracranial compartments. Three of the most well recognized and studied proteins implicated in hydrocephalus are NKCC1, Aquaporin-1 (AQP-1), and Aquaporin-4 (AQP-4). NKCC1 is found in the apical membrane of CPECs, where it can transport sodium, potassium and chloride ions and help drive CSF secretion (Figure 2). NKCC1 is also thought to be responsible for the hypersecretion of CSF in response to inflammation.^33^ It is this protein which is targeted by loop diuretics like furosemide and bumetanide, which are sometimes used in ICP control. Mouse studies have indicated that NKCC1 may be responsible for almost half of CSF production through cotransport of water along with the directional translocation of ions, independent of osmotic effects.^34^ This co-transport of water is thought to occur through the AQP-1 water transporter, which is predominantly, but not exclusively found on the apical surface of CPECs.^35^ Acetazolamide, one of the only other existing pharmaceuticals for managing ICP, acts via a separate pathway through the inhibition of the intracellular enzyme carbonic anhydrase. Acetazolamide has been shown to reduce CSF production in humans by 6 to as much as 50%.^36^

AQP-4, unlike AQP-1, is predominantly located on the basolateral membrane of ependymal cells as well as astrocytic perivascular end feet where it is involved in water transport from the interstitial to intracellular space. AQP-4 null mice have smaller ventricles, decreased CSF production and higher brain water content.^37^ They can also develop hyperpermeability of the BBB, showing that AQP-4 may also be important for BBB integrity.^38^ Although deletion of AQP-4 in AQP-4 null mice as well as small molecule inhibitors of AQP-4 can improve neurological function after cytotoxic cellular edema from pathologies such as ischemic stroke or bacterial meningitis,^39^ worse outcomes are observed in vasogenic models of edema, such as brain tumors.^40^ Given that cytotoxic versus vasogenic edema is caused by an excess of water within the intracellular space versus the interstitial space, respectively, worse outcomes in models of vasogenic edema are presumably due to an inability to selectively clear excess extracellular brain water in mice lacking AQP-4.

### The role of ependymal cells and CPECs

Ependymal cells and CPECs play a critical function in normal CSF dynamics. Directional flow of CSF is generated through the synchronous beating of the cilia of ependymal cells and during development, a steady ependymal flow is required to maintain the patency of the cerebral aqueduct. Cilia not only modulate the directional flow of CSF but may also act as chemoreceptors to regulate the production of CSF.^41^

Not surprisingly then, some of the genes implicated in congenital hydrocephalus in humans function in ciliated ependymal cells and CPECs. In humans with syndromic hydrocephalus, at least 20 genes encoding proteins that are required for either the biosynthesis or proper function of the cilium and/or choroid plexus epithelium have been identified.^42^ However, although many genes have been associated with syndromic forms of hydrocephalus, few are associated with congenital hydrocephalus as a primary or sole feature. These include previously identified mutations in L1 cell adhesion molecular (L1CAM); adaptor-related protein complex 1, sigma-2 subunit (AP1S2); multiple PDZ domain protein (MPDZ); and coiled-coil domain-containing protein 88C (CCDC88C). Mutations in L1CAM, which has been implicated in neuronal migration, axon guidance, and synaptic plasticity,^43^ account for up to 10% of X-linked idiopathic hydrocephalus.^44^ AP1S2 interacts with microtubules to modulate cytoskeletal dynamics,^45^ whereas MPDZ may be important for maintaining tight junctions and the permeability of the choroid plexus epithelium.^46^ CCDC88C acts as a negative regulator of the noncanonical WNT signaling pathway. Interestingly, both the canonical (beta-catenin dependent) and non-canonical Wnt signaling pathways have been implicated in hydrocephalus and play an important role in ependymal cell polarity and ciliary maintenance.^47^, ^48^, ^49^

Many other genes with direct functions in ependymal cells and cilia have also been discovered in animal models of hydrocephalus. In eight of twelve novel autosomal recessive hydrocephalic mutants identified in a study using 4650 knockout mouse lines, the mutation was associated with motile ciliogenesis and ciliary function.^50^ Motile ciliogenesis and maturation of the ependyma depends on the forkhead transcription factor *Foxj1,* and, not surprisingly, *de novo* heterozygous mutations in FOXJ1 cause a motile ciliopathy characterized by hydrocephalus, airway disease, and deficits in right/left asymmetry.^51^

Although ependymal cells and CPECS are modified epithelial cells, they are actually derived from the neuroepithelium or neuroectoderm, which gives rise to cortical neurons and glia.^52^ Consequently, genes that are important for cortical development, specifically the proliferation, migration and maintenance of neural progenitor cells, may also play a role in ependymal and CPEC function and thus hydrocephalus. Indeed, a recent study using whole exome sequencing of 381 patients with sporadic congenital hydrocephalus also identified a cohort of genes which play some role in proliferation and/or differentiation of neural stem cells.^53^ These include *de novo* mutations in TRIM71, SMARCC1, PTCH1 and SHH. The authors conclude that genes associated with congenital hydrocephalus typically fall into one of two categories; those involved in CSF circulation, or those involved in normal cortical development.

As a whole, therefore, many of the genes associated with both congenital and syndromic hydrocephalus in both animal models and humans play a role in the development, maintenance, and/or function of ciliated ependymal cells and/or CPECs. Furthermore, many of these genes may overlap between CPECs, ependyma and neural progenitors, with abnormalities of the ependyma and choroid plexus epithelium correlating with changes in the adjacent subventricular zone, and vice versa.

### Ependymal and CPECs and the role of inflammation and iron

Although the examples discussed above are associated with forms of congenital hydrocephalus, perturbations of normal CSF ependymal and CPEC ciliary function also occur in models of secondary, acquired hydrocephalus. For example, there is evidence that post hemorrhagic and post infectious hydrocephalus may cause an inflammatory reaction which impairs normal ependymal and choroid plexus function. The presence of foreign inhabitants of CSF (either viral, bacterial or fungal) often sets off a non-specific inflammatory response, including the release of cytokines such as Il-1β, IL-6 and TNF-α, as well as local immunoglobulin production by plasma cells.^54^ Animal studies have shown that intraventricular injection of autologous blood into the lateral ventricles is sufficient to produce ventriculomegaly and activate NF-kB and cytokine production in choroid plexus epithelium, which corresponds to a more than three-fold increase in CSF production.^55^ In the model proposed by Dr. Kahle and colleagues, CSF hypersecretion occurs acutely in the first 7 days after infection or hemorrhage and is then followed by scarring and inflammation resulting in CSF malabsorption. The TLR4-NF-κB pathway is a critical mediator of the innate immune response, and inhibition of TLR4 signaling attenuated ventriculomegaly as well as markers of ependymal inflammation in post hemorrhagic models of hydrocephalus.^56^ Consistent with the idea that acquired hydrocephalus may be partially immune mediated, incidences of hydrocephalus were significantly lower in a cohort of patients who received dexamethasone after subarachnoid hemorrhage.^57^

Intraventricular injection of autologous whole blood also causes a buildup of iron in the CSF. Iron has also been shown to play a distinct role in hydrocephalus and may cause direct damage to ependymal cilia. Normal ependymal cells take up iron from the CSF and prevent its diffusion to the rest of the brain; consequently, levels of iron can accumulate in brain parenchyma if ependymal cells are damaged. In turn, systemic deferoxamine treatment has been found to partially reverse this iron accumulation and alleviate hydrocephalus in a rat model of IVH.^58^

## Conclusions and future directions

Altogether, hydrocephalus is one of the most common yet least well understood clinical entities in neurosurgical practice. Our methodology for the treatment of hydrocephalus has also not changed significantly since the 1950s, when the first silicone ventriculoperitoneal shunt (VPS) was placed by Richard Ames.^59^ Moreover, shunting systems for the treatment of hydrocephalus frequently fail, leading to multiple hospitalizations and/or surgeries, significant loss of health care dollars, and reduced quality of life. The purpose of this review article was to highlight recent research discoveries regarding the genetic and mechanistic determinants of hydrocephalus, with a focus on the genetic and molecular factors underpinning the development of congenital and acquired hydrocephalus.

Although clinical focus has been on treating hydrocephalus once it occurs, understanding the mechanisms by which this entity develops may help us to identify novel therapeutic strategies. Moreover, the model of a unidirectional CSF flow system from the ventricles to the subarachnoid space and uptake via arachnoid granulations is overly simplistic. CSF can permeate all brain compartments, and perivascular networks may play a much more important role than previously recognized. In fact, “glymphatic dysfunction” is being recognized as a hallmark of many different pathological processes, including sleep disturbances and psychiatric conditions such as major depressive disorder.^60^ A more comprehensive understanding of how CSF moves throughout the brain and the development of novel methods to modulate these pathways may thus be applicable to numerous brain disorders, from neurodegenerative diseases to psychiatric illnesses and brain tumors.^61^

Ependymal cells and CPECs also play a variety of key roles in both congenital and acquired hydrocephalus. In addition to maintaining directional CSF flow through motile cilia, ependymal cells direct transport of ions and solutes in the surrounding intraventricular and parenchymal compartments, the production of growth factors that may be important for neurogenic proliferation and survival, and the choroid plexus epithelium acts as a barrier at the CSF-brain interface, maintaining cell junctions at the choroid plexus. Both ependyma and CPECs can contribute to both genetic causes of hydrocephalus (via their role in normal brain development) and to acquired causes, as they are subject to functional impairment after any secondary injury that causes associated inflammatory responses and damage.

Given the limitations and complications associated with shunt systems, a therapeutic strategy targeting the genes and/or proteins involved in the development of hydrocephalus is a promising alternative solution. Choroid plexus and ependyma are especially suitable targets for therapeutic interventions including viral, RNA or protein based therapies given their location as the cells directly lining CSF spaces in the brain and spinal cord. For example, AAV vectors have been shown to produce strong long-term expression within the choroid plexus and ependyma in mouse models,^62^ and human trials for intrathecal recombinant protein delivery as well as AAV based enzyme replacement therapy using AAV viruses are well underway.^63^

In order to identify these novel therapeutic strategies, however, it is first essential to understand the molecular mechanisms underlying the pathogenesis of hydrocephalus. In deciphering these key regulators of hydrocephalus at a genetic and molecular level, the ultimate goal is to develop novel therapies to modify the function of ependymal and CPECs and ultimately abrogate the need for ventricular shunting. It will be especially exciting to see how our understanding of CSF dynamics and hydrocephalus changes over the coming years, and how this will come to influence future neurosurgical practice.

### Outstanding questions

Many questions about CSF physiology and hydrocephalus remain to be addressed. First and foremost, what is the purpose of CSF reservoirs? What are the key molecular pathways that regulate the brain's response to changes in intracranial pressure? How does the choroid plexus respond to these changes and what are the molecular cues that govern the regulation of CSF production? How can we manipulate the functions of choroid plexus and ependymal cells to prevent and/or treat patients with hydrocephalus? Once we learn the answers to some of these fundamental questions, we may begin to tackle the problem of hydrocephalus and its association to the most common cerebral pathologies such as trauma, brain hemorrhages, stroke, neurodegenerative disease and even abnormal ageing.

### Search strategy and selection criteria

References for this review were identified by searches on both Pubmed and Google Scholar from 1960 to June 2021 and references from relevant articles. The search terms “hydrocephalus”, “glymphatic system”, “cerebrospinal fluid dynamics”, “cerebrospinal fluid physiology”, cerebrospinal fluid evolution”, and “hydrocephalus mechanisms” were used. There were no language restrictions. The final reference list was based on relevance to the topics covered in the review.

### Author contribution statement

AB conceived of the topic of the review, performed the literature searches and wrote the manuscript. PF, KK, and EA contributed to intellectual development and helped to edit the final manuscript. All authors read and approved the final version of the manuscript

## Declaration of Interests

There are no conflicts of interest to declare.

## References

[1] Aschoff A., Kremer P., Hashemi B., Kunze S. (1999). The scientific history of hydrocephalus and its treatment. Neurosurg Rev.

[2] Brocklehurst G. (1979). The significance of the evolution of the cerebrospinal fluid system. Ann R Coll Surg Engl.

[3] Guthery F.S., Beasom S.L., Jones L. (1979). Cerebrospinal nematodiasis caused by Parelaphostrongylus tenuis in Angora goats in Texas. J Wildl Dis.

[4] Desmond M.E., Jacobson A.G. (1977). Embryonic brain enlargement requires cerebrospinal fluid pressure. Dev Biol.

[5] Chang J.T., Sive H. (2012). Manual drainage of the zebrafish embryonic brain ventricles. J Vis Exp.

[6] Ryann F. (2016). Lehtinen maria emergence and developmental roles of the cerebrospinal fluid system. Developmental Cell.

[7] Bueno D., Garcia-Fernandez J. (2016). Evolutionary development of embryonic cerebrospinal fluid composition and regulation: an open research field with implications for brain development and function. Fluids Barriers CNS.

[8] Praetorius J., Damkier H.H. (2017). Transport across the choroid plexus epithelium. Am J Physiol Cell Physiol.

[9] Bothwell S.W., Janigro D., Patabendige A. (2019). Cerebrospinal fluid dynamics and intracranial pressure elevation in neurological diseases. Fluids Barriers CNS.

[10] Dailey M.E. (1931). The equillibrium between cerebrospinal fluid and blood plasma. JBC.

[11] Tamune H., Takeya H., Suzuki W. (2014). Cerebrospinal fluid/blood glucose ratio as an indicator for bacterial meningitis. Am J Emerg Med.

[12] Viallon A., Botelho-Nevers E., Zeni F. (2016). Clinical decision rules for acute bacterial meningitis: current insights. Open Access Emerg Med.

[13] Ghaffari H., Grant S.C., Petzold L.R., Harrington M.G. (2020). Regulation of CSF and brain tissue sodium levels by the blood-CSF and blood-brain barriers during migraine. Front Comput Neurosci.

[14] Masvekar R., Phillips J., Komori M., Wu T., Bielekova B. (2021). Cerebrospinal fluid biomarkers of myeloid and glial cell activation are correlated with multiple sclerosis lesional inflammatory activity. Front Neurosci.

[15] Katayama T., Sawada J., Takahashi K., Yahara O. (2020). Cerebrospinal fluid biomarkers in Parkinson's disease: a critical overview of the literature and meta-analyses. Brain Sci.

[16] Obrocki P., Khatun A., Ness D. (2020). Perspectives in fluid biomarkers in neurodegeneration from the 2019 biomarkers in neurodegenerative diseases course-a joint PhD student course at University College London and University of Gothenburg. Alzheimers Res Ther.

[17] Taghdiri F., Gumus M., Algarni M., Fasano A., Tang-Wai D., Tartaglia M.C. (2020). Association between cerebrospinal fluid biomarkers and age-related brain changes in patients with normal pressure hydrocephalus. Sci Rep.

[18] Roales-Bujan R., Paez P., Guerra M. (2012). Astrocytes acquire morphological and functional characteristics of ependymal cells following disruption of ependyma in hydrocephalus. Acta Neuropathol.

[19] Narita K., Takeda S. (2015). Cilia in the choroid plexus: their roles in hydrocephalus and beyond. Front Cell Neurosci.

[20] Nonami Y., Narita K., Nakamura H., Inoue T., Takeda S. (2013). Developmental changes in ciliary motility on choroid plexus epithelial cells during the perinatal period. Cytoskeleton.

[21] Brinker T., Stopa E., Morrison J., Klinge P. (2014). A new look at cerebrospinal fluid circulation. Fluids Barriers CNS.

[22] Shigeno S., Andrews P.L.R., Ponte G., Fiorito G. (2018). Cephalopod brains: an overview of current knowledge to facilitate comparison with vertebrates. Front Physiol.

[23] Abbott N.J. (2004). Evidence for bulk flow of brain interstitial fluid: significance for physiology and pathology. Neurochem Int.

[24] Mack J., Squier W., Eastman J.T. (2009). Anatomy and development of the meninges: implications for subdural collections and CSF circulation. Pediatr Radiol.

[25] Chen C.C., Liu C.L., Tung Y.N. (2011). Endoscopic surgery for intraventricular hemorrhage (IVH) caused by thalamic hemorrhage: comparisons of endoscopic surgery and external ventricular drainage (EVD) surgery. World Neurosurg.

[26] Mollanji R., Bozanovic-Sosic R., Zakharov A., Makarian L., Johnston M.G. (2002). Blocking cerebrospinal fluid absorption through the cribriform plate increases resting intracranial pressure. Am J Physiol Regul Integr Comp Physiol.

[27] Iliff J.J., Wang M., Liao Y. (2012). A paravascular pathway facilitates CSF flow through the brain parenchyma and the clearance of interstitial solutes, including amyloid beta. Sci Transl Med.

[28] Louveau A. (2015). [Cerebral lymphatic drainage: implication in the brain immune privilege]. Med Sci.

[29] Ahn J.H., Cho H., Kim J.H. (2019). Meningeal lymphatic vessels at the skull base drain cerebrospinal fluid. Nature.

[30] Bu Y., Chen M., Gao T., Wang X., Li X., Gao F. (2016). Mechanisms of hydrocephalus after intraventricular haemorrhage in adults. Stroke Vasc Neurol.

[31] Tomycz L.D., Hale A.T., George T.M. (2017). Emerging insights and new perspectives on the nature of hydrocephalus. Pediatr Neurosurg.

[32] Mestre H., Tithof J., Du T. (2018). Flow of cerebrospinal fluid is driven by arterial pulsations and is reduced in hypertension. Nat Commun.

[33] Karimy J.K., Zhang J., Kurland D.B. (2017). Inflammation-dependent cerebrospinal fluid hypersecretion by the choroid plexus epithelium in posthemorrhagic hydrocephalus. Nat Med.

[34] Steffensen A.B., Oernbo E.K., Stoica A. (2018). Cotransporter-mediated water transport underlying cerebrospinal fluid formation. Nat Commun.

[35] Owler B.K., Pitham T., Wang D. (2010). Aquaporins: relevance to cerebrospinal fluid physiology and therapeutic potential in hydrocephalus. Cerebrospinal Fluid Res.

[36] Wall M., McDermott M.P., Committee NIIHSGW (2014). Effect of acetazolamide on visual function in patients with idiopathic intracranial hypertension and mild visual loss: the idiopathic intracranial hypertension treatment trial. JAMA.

[37] Li X., Kong H., Wu W., Xiao M., Sun X., Hu G. (2009). Aquaporin-4 maintains ependymal integrity in adult mice. Neuroscience.

[38] Zhou J., Kong H., Hua X., Xiao M., Ding J., Hu G. (2008). Altered blood-brain barrier integrity in adult aquaporin-4 knockout mice. Neuroreport.

[39] Farr G.W., Hall C.H., Farr S.M. (2019). Functionalized phenylbenzamides inhibit aquaporin-4 reducing cerebral edema and improving outcome in two models of CNS injury. Neuroscience.

[40] Verkman A.S., Binder D.K., Bloch O., Auguste K., Papadopoulos M.C. (2006). Three distinct roles of aquaporin-4 in brain function revealed by knockout mice. Biochim Biophys Acta.

[41] Narita K., Kawate T., Kakinuma N., Takeda S. (2010). Multiple primary cilia modulate the fluid transcytosis in choroid plexus epithelium. Traffic.

[42] Banizs B., Pike M.M., Millican C.L. (2005). Dysfunctional cilia lead to altered ependyma and choroid plexus function, and result in the formation of hydrocephalus. Development.

[43] Itoh K., Fushiki S. (2015). The role of L1cam in murine corticogenesis, and the pathogenesis of hydrocephalus. Pathol Int.

[44] Adle-Biassette H., Saugier-Veber P., Fallet-Bianco C. (2013). Neuropathological review of 138 cases genetically tested for X-linked hydrocephalus: evidence for closely related clinical entities of unknown molecular bases. Acta Neuropathol.

[45] Saillour Y., Zanni G., Des Portes V. (2007). Mutations in the AP1S2 gene encoding the sigma 2 subunit of the adaptor protein 1 complex are associated with syndromic X-linked mental retardation with hydrocephalus and calcifications in basal ganglia. J Med Genet.

[46] Yang J., Simonneau C., Kilker R. (2019). Murine MPDZ-linked hydrocephalus is caused by hyperpermeability of the choroid plexus. EMBO Mol Med.

[47] Zhang J., Chandrasekaran G., Li W. (2020). Wnt-PLC-IP3-Connexin-Ca(2+) axis maintains ependymal motile cilia in zebrafish spinal cord. Nat Commun.

[48] Ohata S., Nakatani J., Herranz-Perez V. (2014). Loss of Dishevelleds disrupts planar polarity in ependymal motile cilia and results in hydrocephalus. Neuron.

[49] Xing L., Anbarchian T., Tsai J.M., Plant G.W., Nusse R. (2018). Wnt/beta-catenin signaling regulates ependymal cell development and adult homeostasis. Proc Natl Acad Sci U S A.

[50] Vogel P., Read R.W., Hansen G.M. (2012). Congenital hydrocephalus in genetically engineered mice. Vet Pathol.

[51] Wallmeier J., Frank D., Shoemark A. (2019). De novo mutations in FOXJ1 result in a motile ciliopathy with hydrocephalus and randomization of left/right body asymmetry. Am J Hum Genet.

[52] Liddelow S.A. (2015). Development of the choroid plexus and blood-CSF barrier. Front Neurosci.

[53] Jin S.C., Dong W., Kundishora A.J. (2020). Exome sequencing implicates genetic disruption of prenatal neuro-gliogenesis in sporadic congenital hydrocephalus. Nat Med.

[54] Benninger F., Steiner I. (2017). CSF in acute and chronic infectious diseases. Handb Clin Neurol.

[55] Simard P.F., Tosun C., Melnichenko L., Ivanova S., Gerzanich V., Simard J.M. (2011). Inflammation of the choroid plexus and ependymal layer of the ventricle following intraventricular hemorrhage. Transl Stroke Res.

[56] Wang Y.C., Wang P.F., Fang H., Chen J., Xiong X.Y., Yang Q.W. (2013). Toll-like receptor 4 antagonist attenuates intracerebral hemorrhage-induced brain injury. Stroke.

[57] Schurkamper M., Medele R., Zausinger S., Schmid-Elsaesser R., Steiger H.J. (2004). Dexamethasone in the treatment of subarachnoid hemorrhage revisited: a comparative analysis of the effect of the total dose on complications and outcome. J Clin Neurosci.

[58] Chen Z., Gao C., Hua Y., Keep R.F., Muraszko K., Xi G. (2011). Role of iron in brain injury after intraventricular hemorrhage. Stroke.

[59] Weisenberg S.H., TerMaath S.C., Seaver C.E., Killeffer JA. (2016). Ventricular catheter development: past, present, and future. J Neurosurg.

[60] Seo J.S., Mantas I., Svenningsson P., Greengard P. (2021). Ependymal cells-CSF flow regulates stress-induced depression. Mol Psychiatry.

[61] Liu X., Gao C., Yuan J. (2020). Subdural haematomas drain into the extracranial lymphatic system through the meningeal lymphatic vessels. Acta Neuropathol Commun.

[62] Yamazaki Y., Hirai Y., Miyake K., Shimada T. (2014). Targeted gene transfer into ependymal cells through intraventricular injection of AAV1 vector and long-term enzyme replacement via the CSF. Sci Rep.

[63] Sevin C., Deiva K. (2021). Clinical trials for gene therapy in lysosomal diseases with CNS involvement. Front Mol Biosci.

[64] Jones H.C. (1979). Comparative aspects of the cerebrospinal fluid systems in vertebrates. Sci Prog.

